# Rays from the Eye

**Published:** 1921-09-17

**Authors:** 


### RAYS FROM THE EYE.

Dr. Charles Russ has demonstrated a very interesting and puzzling phenomenon. He has demonstrated that under certain conditions a solenoid suspended horizontally by a silk thread will rotate under the gaze of a human eye, and will rotate in either direction according to the end gazed at, as if under the repellent impact of rays from the eye. He has made experiments eliminating all forces, such as heat, which might account for the rotation, and still the solenoid rotates under the glance of the eye. It seems, therefore, possible that electric or other waves emanate from the eye, and it is even possible that some new form of energy is involved; but even on such assumptions there are many difficulties to surmount before a complete explanation of the phenomenon can be formulated.

Dr. Russ's machine has had its predecessors. Not so many years ago a cute palmist used to give exhibitions of a machine—a "will-register" he called it—said to have been invented by a French doctor, in which a needle was alleged to revolve under the influence of the will. The needle certainly did revolve, and apparently Gladstone and some other well-known men were impressed if not duped by it. But this remarkable instrument seems to have been suddenly afflicted with modesty, for it has withdrawn from the public gaze, and we hear no more of it.

This spurious will-register, of course, must not be put on the same footing as Dr. Russ's solenoid jar; but another instrument, invented in 1887 by Monsieur J. Thore, was a worthier predecessor. M. Thore found that cylinders made of ivory and other materials, and suspended by a silk thread, rotated when approached by an observer. He considered light, heat, electricity, magnetism, gravity, and air currents all inadequate to explain the rotation, and came to the conclusion that some force emanated from the observer. He made numerous ingenious experiments to prove that heat was not the cause of the phenomenon, such as interposing metallic vessels full of boiling water between the cylinder and observer, and altogether conducted his observations in a thoroughly scientific way. But Sir William Crookes carefully and thoroughly investigated the matter and showed that the rotation was due to molecular bombardment caused by the heat of the observer's body, and that a bottle of hot water was equally successful in causing the cylinder to revolve.

Whether Dr. Russ's results can be explained by forces already known or whether they are indications of a new force remains to be seen. His own exposition of his discovery has been guarded, modest, and scientific, and the phenomenon he has discovered is at least interesting and mysterious, and requires explanation.

